# Supplementary material for: Efficient Retention and Alpha Spectroscopy of Actinides from Aqueous Solutions Using a Combination of Water-Soluble Star-like Polymers and Ultrafiltration Membranes
Source: Polymers (Basel). 2022 Aug 23;14(17):3441. doi: 10.3390/polym14173441 (PMC9460127; doi:10.3390/polym14173441)
Supplement: Supplementary file 1 [file polymers-14-03441-s001.zip › polymers-1838043-supplementary.pdf]

Supplementary Materials

Efficient retention and alpha spectroscopy of actinides from aqueous solutions using a combination of water-soluble star-like polymers and ultrafiltration membranes

Valery N. Bliznyuk[1†*], Nataliya V. Kutsevol[2], Yuliia I. Kuziv[2], Scott M. Husson[3†], Timothy A. DeVol[1†]

[1] *Environmental Engineering and Earth Sciences, Clemson University, Anderson, SC 29625, USA*

[2] *Faculty of Chemistry, Taras Shevchenko National University of Kyiv, 60 Volodymyrska Street, 01601 Kyiv, Ukraine*

[3] *Chemical and Biomolecular Engineering, Clemson University, Clemson, SC 29634, USA*

[†] *Center for Nuclear Environmental Engineering Sciences and Radioactive Waste Management (NEESRWM) at Clemson University, Clemson SC 29634-0942 USA*

Table S1. The molecular parameters of star-like polymers DXX-PAAm.

**Sample**	**M_w×10^{-5}, g/mol**	**R_z, nm**
D20-PAA	8.5	60
D70-PAA	7.9	52
D500-PAA	13.6	67

Table S2 Composition of ^{242}Pu stock solution

Isotope	Mass Fraction	Specific Activity [Ci/g]	Sample activity concentration [Ci/g]	Activity ratios normalized to Pu-242	Type of decay, Eα [MeV] f(%)
**Pu-238**	0.003	1.70E+01	5.10E-04	0.13	5.499 (71), 5.456 (29)
**Pu-239**	0.005	6.20E-02	3.10E-06	7.95E-04	5.157 (71), 5.144 (17), 5.105 (12)
**Pu-240**	0.022	2.30E-01	5.06E-05	1.30E-02	5.168 (73), 5.124 (27)
**Pu-241**	0.006	1.00E+02	6.00E-03	1.54	β- decay
**Pu-242**	99.962	3.90E-03	3.90E-03	1.00	4.902 (76.5), 4.858 (23.5)
**Pu-244**	0.002	1.80E-05	3.60E-10	9.23E-08	4.589 (80.5), 4.546 (19.4)

PuO_2 solid we obtained from Oak Ridge in nitric acid (date 02/28/1980).
The amounts of ^{241}Pu and ^{238}Pu could significantly change since then.
^{241}Pu decays to ^{241}Am and then to ^{237}Np.
^{237}Np α energy: 4.788 MeV (47.64%), 4.771 MeV (23.2%), 47665 MeV (9.3%), 4.640 MeV (6.43%), 4.665 MeV (3.45%).
^{241}Am α energy: 5.486 MeV (84%), 5.443 MeV (13%)

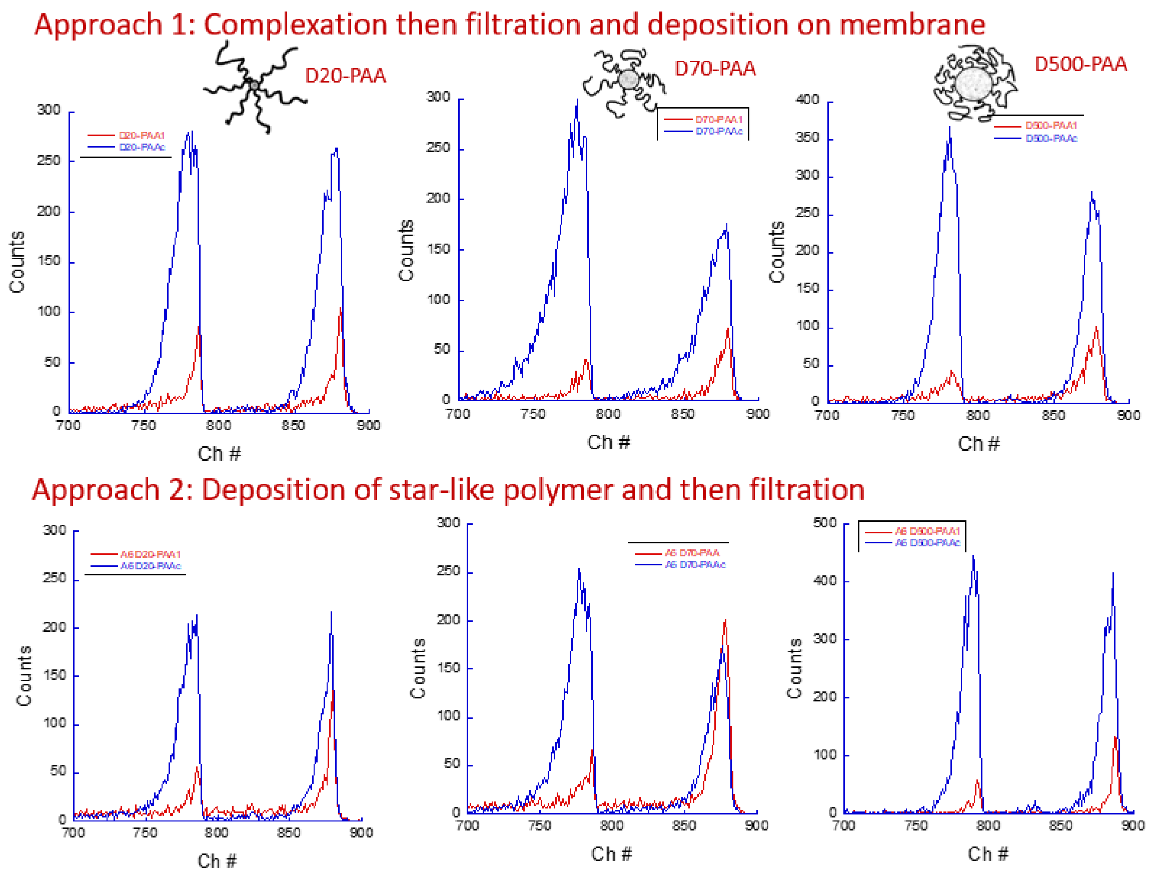

Figure S1. Alpha spectra of PVDF A6 membranes with polyacrylamide-Pu complexes during adsorption in Approach 1 and Approach 2.

Table S3. Calculated ratios of integral intensities of two characteristic peaks in alpha spectra of dextran-PAA films with adsorbed Pu from aqueous solution (**Figure S1**). Comparison between two approaches and neutral vs ionic forms of the PAA shell as three different dextran core sizes used in this study.

Dextran core size	$I_{Pu\text{-}242}/I_{Am\text{-}241}$			
	Approach 1		Approach 2	
	Neutral PAA	Ionic PAAc	Neutral PAA	Ionic PAAc
D20-PAA	0.9	1.25	0.77	1.6
D70-PAA	0.7	1.97	0.45	1.85
D500-PAA	0.56	1.4	0.5	1.3

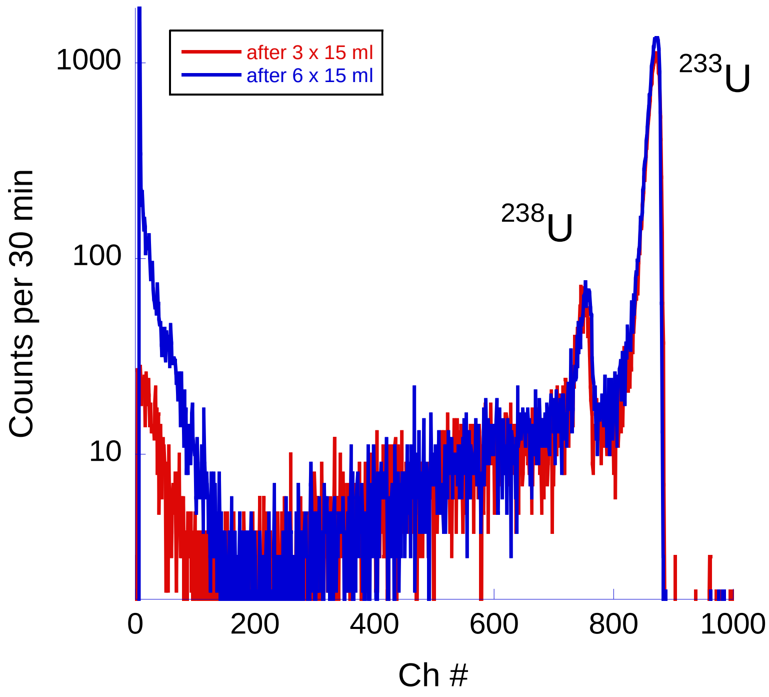

Figure S2. Alpha spectra of PVDF A6 membrane coated with D500-PAAc (1 mg) polymer after filtration of 3 x 15 ml and 6 x 15 ml volumes of 25 ppm U solutions. The membrane was dried after each filtration step in air and was additionally dried in vacuum after 3 and after 6 steps for alpha spectroscopy experiments.

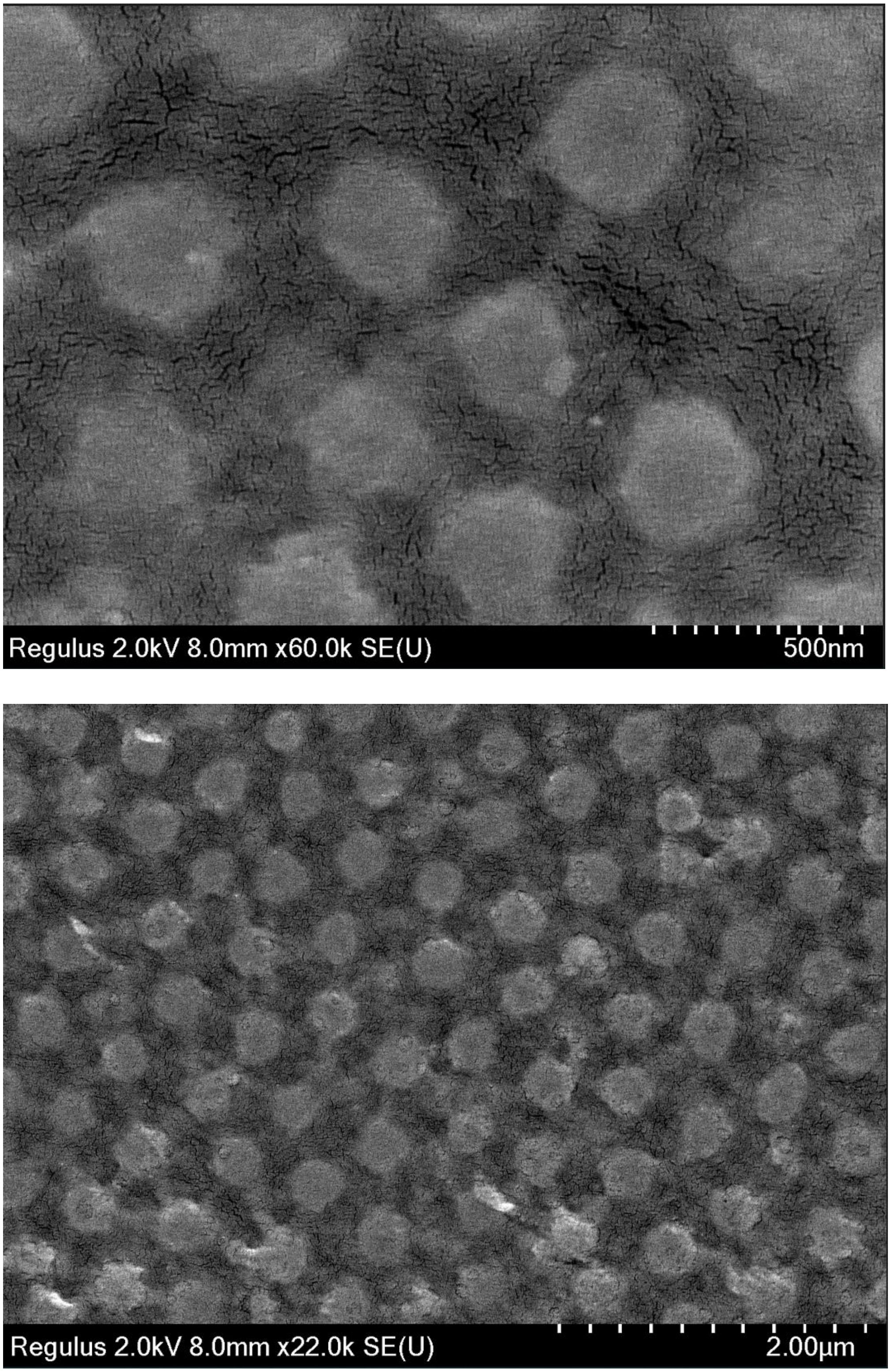

Figure S3. SEM images of supramolecular self-organization in D500-PAA film formed during pressure-assisted deposition on A6 PVDF UF membrane from water solution (Approach 1).
